# *BitBrain* and Sparse Binary Coincidence (SBC) memories: Fast, robust learning and inference for neuromorphic architectures

**DOI:** 10.3389/fninf.2023.1125844

**Published:** 2023-03-21

**Authors:** Michael Hopkins, Jakub Fil, Edward George Jones, Steve Furber

**Affiliations:** Advanced Processor Technologies Group, Department of Computer Science, The University of Manchester, Manchester, United Kingdom

**Keywords:** single-pass learning, neuromorphic, efficient inference, classification, machine learning, robust, event-based, IoT

## Abstract

We present an innovative working mechanism (the *SBC memory*) and surrounding infrastructure (*BitBrain*) based upon a novel synthesis of ideas from sparse coding, computational neuroscience and information theory that enables fast and adaptive learning and accurate, robust inference. The mechanism is designed to be implemented efficiently on current and future neuromorphic devices as well as on more conventional CPU and memory architectures. An example implementation on the SpiNNaker neuromorphic platform has been developed and initial results are presented. The SBC memory stores coincidences between features detected in class examples in a training set, and infers the class of a previously unseen test example by identifying the class with which it shares the highest number of feature coincidences. A number of SBC memories may be combined in a *BitBrain* to increase the diversity of the contributing feature coincidences. The resulting inference mechanism is shown to have excellent classification performance on benchmarks such as MNIST and EMNIST, achieving classification accuracy with single-pass learning approaching that of state-of-the-art deep networks with much larger tuneable parameter spaces and much higher training costs. It can also be made very robust to noise. *BitBrain* is designed to be very efficient in training and inference on both conventional and neuromorphic architectures. It provides a unique combination of single-pass, single-shot and continuous supervised learning; following a very simple unsupervised phase. Accurate classification inference that is very robust against imperfect inputs has been demonstrated. These contributions make it uniquely well-suited for edge and IoT applications.

## 1. Introduction

With the inevitable ubiquity of AI (Artificial Intelligence) decision-making that the IoT (Internet of Things) will facilitate, there is a clear need for mechanisms and architectures that allow accurate inferences to be made quickly, robustly (in the presence of imperfect input data) and with low energy use. Current state of the art information architectures such as deep learning can provide excellent inference but at the cost of vast numbers of parameters that need to be learned at training time and computed at inference time. This makes their learning phase a huge resource commitment in both energy and time, and discourages a local implementation of inference at the end of realistic network branches where storage, energy and communication resources are likely to be severely constrained. Arguably, their requirement for huge parameter sets also makes them liable to over-fitting and the lack of robustness that this leads to, leading some researchers to use *ad hoc* methods such as dropout which can sometimes improve performance but at the cost of a further significant learning burden. It is also currently not clear how well they can be applied to the problem of continuous learning which is likely to become more important in practical applications.

In the near future, intelligent decisions and classifications are going to become required in an increasing number of devices and architectures with constrained resources. This will focus attention on the following issues, and technologies that help to address them will become desirable:

- Faster learning and inference.
- Energy-efficient learning and inference.
- More robust inference in the presence of imperfections and noise at the network inputs and partial system errors so that performance degradation is graceful rather than brittle.
- An easy mapping onto the growing number of neuromorphic architectures that facilitate large gains in speed and energy efficiency.
- A natural receiving mechanism for event-based visual and audio sensors which leverage further gains in energy use and communication bandwidth.

These technologies should be of interest to anyone who wants to address the issues described above, in particular those looking to make fast and reliable inferences at the edge. Another possibility is those who would like to create a large number of small and efficient self-contained inference modules which perform potentially complex pattern recognition with a very low energy-latency product and communicate *via* relatively simple and sparse messages. The latter is a good conceptual match to the dendritic computation paradigm in computational neuroscience which is gaining traction, and which changes quite significantly the balance between computation and communication in large neural networks where each action potential now becomes a carrier of more information content.

We introduce an innovative working mechanism (the *SBC memory*) and surrounding infrastructure (*BitBrain*) based upon a novel synthesis of ideas from sparse coding, computational neuroscience and information theory.^1^ The key contributions of this technology presented here are:

- Single-pass and single-shot supervised learning; following a simple unsupervised phase where parameters are learned quickly in a simple and “local” way that does not require global optimisation over high-dimensional spaces or the calculation of derivatives.
- Accurate inference (currently classification) that is very robust against imperfect inputs.
- Simple support for continuous adaptive learning.
- Algorithms that are designed to be implemented with excellent energy efficiency on conventional CPUs and memory architectures, and on current and future neuromorphic devices.
- A natural target for the increasing number of event-based sensors such as silicon retinas, enabling further energy and bandwidth gains to be exploited—in particular for edge computing and IoT devices.

## 2. Background

The ideas that contributed to the *BitBrain* mechanism are drawn from a variety of areas:

### 2.1. Sparsity of activity and homeostasis

An aspect of neural activity which is clear in the neocortex and also globally to some extent is that activity—in terms of action potentials at least—is relatively sparse. Perhaps 5–10% of neurons are firing in a specific time window. If one considers the massive complexity of the brain and all its interconnections then this surely must imply some kind of self-adaption or self-regulation, and for this to work consistently one can further infer that it should be present at the local level. Hence we believe that some form of homeostasis in the basic functional mechanisms is an important part of any neural or neurally-inspired mechanism. In this context, homeostasis means that the underlying physiological mechanisms tend toward a natural, equilibrium rate of activity despite all the complex non-linearities and interactions that they share.

Sparse memory mechanisms have been discussed before. One important example is the concept of Sparse Distributed Memory (SDM) introduced by Pentti Kanerva in his PhD thesis (Kanerva, 1988). The key concept in an SDM is to use random address decoders to map a binary input space into a very high-dimensional intermediate space where associated information can be stored very sparsely and redundantly, leading to robust recovery of that information whenever a similar input is presented to the memory. Kanerva speculated that a similar mechanism might be at work in the cerebellum, where there are similarities between the neuronal organisation and the structure of an SDM. Furber et al. developed this idea further showing that the same approach was effective using sparse *N-of-M* codes for both the input and the stored information (Furber et al., 2004) and an SDM could even be used to store and recover the temporal patterns (Furber et al., 2007) when rank-order codes (Thorpe and Gautrais, 1998) were used.

There are similarities between the address decoders used in our SBCs and those used in SDMs, though we have added homeostatic tuning mechanisms such as threshold adjustment and structural plasticity to the original address decoder concept.

### 2.2. Robustness in the presence of noise, errors, or partial failure

Biological neural systems are extremely good at inferring correct decisions and actions from imperfect sources of information, whether this is poor data from sensory systems in noisy or otherwise perturbed environments, imperfect conditions within the neural mechanism itself (such as the presence of alcohol or other disorders of the ideal equilibrium) or a total failure of some parts of the system.

Neurons and synapses are far from perfect processing elements, with probabilistic and somewhat unreliable transfer functions even when working at their full potential, and prone to change and failure as are all biological mechanisms. Yet in the presence of all these perturbations the system as a whole performs very well. Understanding how this apparent paradox can be explained will be an important step forwards in engineering mechanisms of inference that degrade gracefully in the presence of realistic amounts of error and uncertainty in their working environment. In the 1980s, inspired partly by the thinking of the Japanese engineer Genichi Taguchi, much research and practical work focused on the importance of this issue in engineering robust systems (Phadke, 1989; Edwards et al., 2000) and the outcome was a change of focus in the design process which remains current today.

It has also been suggested that noise and uncertainty within the processing mechanism is not a problem but is in fact a valuable resource in allowing this robustness to occur (Maass, 2014) and this is a view that we agree with.

### 2.3. Avoiding optimisation over high-dimensional parameter spaces

Optimisation over high-dimensional parameter spaces with multiple non-linear objective functions where there are vast numbers of local optima is very hard to do consistently well, and intuitions about dimensions above about 10 do not serve well in realistic problems. When one considers that in some contemporary deep neural networks (DNNs) the learning mechanism is optimising an error function over perhaps billions or trillions of parameters, one can see that both the energy and time cost, and the almost guaranteed sub-optimality that will result, are major issues. That is to say nothing of the inevitability of over-fitting, which is clear from probability and information theory when the numbers of independent degrees of freedom in the system may be several orders of magnitude higher than required for the appropriate model (see for example, chapter 4 in both Sivia and Skilling, 2006 and Jaynes, 2003 books). As well as being too numerous these degrees of freedom are rarely if ever, apportioned where they are most justified within the resulting very complex models though work has been done on how to approach this problem on a rational basis (Tishby et al., 1999).

### 2.4. Spike-time coding, dendritic computation, and local unsupervised learning

There has been a long history of debate about the coding mechanisms used in brains to represent and transmit information. The main two contenders at the base computational level are spike-time and rate coding, though these apparently distinct categories can overlap somewhat at the extremes of their ranges (Reike et al., 1996). For output neurons that drive muscles there is general agreement that rate coding is used, however within more time-critical and energy-sensitive parts of the brain many believe that spike-time coding must be involved. This opens up many avenues of exploration for the kinds of representations and learning mechanisms that generate the sparse activity that we observe, whilst at the same time allowing fast and energy-efficient computation.

It is clear that neural systems do at least some of their learning (i) locally and (ii) without reference to a global error or utility function. This is presumably to help the mechanism as a whole orient itself in relation to the representations required in order to solve the higher-level problem using the minimum amount of time and energy. In machine learning terms we can say this is *unsupervised learning*. A proven neurally-inspired mechanism for facilitating this is spike-time dependent plasticity (STDP), but the decision about where to access the required post-synaptic signal can be debated. Many implementations choose to use the action potential at the soma after having back-propagated through the dendritic tree, but there are some issues with both timing and reliability in this model. With the recent increased interest in dendritic computation (London and Häusser, 2005; Stuart et al., 2016) it has become apparent that NMDA (N-methyl-D-aspartate) potentials local to the synapses involved (i.e., within the local synaptic cluster or at least in a close part of the dendritic branch) can provide the signal required without these issues (Larkum and Nevian, 2008; Branco and Häusser, 2010; Govindarajan et al., 2011) and some work has already been done to apply these understandings to neuromorphic architectures (Yang et al., 2021a). There are many other aspects of dendritic computation which may elucidate mechanisms that allow for sparse and robust representations which balance local and global behaviours (Mel, 1992; Papoutsi et al., 2014; Kastellakis et al., 2015; Ahmad and Hawkins, 2016; Richards and Lillicrap, 2019).

Although adjusting the size of local synapses and hence their drive capability is often the chosen mechanism for STDP, we instead choose structural plasticity as an effective mechanism so that synapses are added or removed using a Hebbian approach (Hopkins et al., 2018) where synapse size only relates to its longevity. This allows us to stay with binary computation and connectivity in order to stay consonant with some of the other aims outlined in this section.

### 2.5. Low resolution computation and mapping onto neuromorphic substrates

In neural systems memory and computation are colocated, setting it apart from the von Neumann model. The ideas inherent in neuromorphic computation should help us to understand how biological neural systems achieve their remarkable performance and energy-efficiency. Such mechanisms should take advantage of engineering opportunities for: energy efficiency, sparsity of activity, low resolution (ideally binary) computation and communication, massive parallelism, asynchrony and event-driven computation. Choices made at the algorithmic design stage can facilitate this mapping onto current and future substrates.

Contemporary large-scale machine learning displays a strong trend toward lower-resolution parameters and computation to leverage the large gains in energy and storage efficiency that result. The lowest useful resolution is the single bit. Earlier work has taken similar directions, though at the time probably for different reasons. Random Access Memory (RAM)-based methods for machine learning have been around since the late 1950s. Their direct use of RAM for storing the inference mechanism based upon complex patterns of binary logic learned directly from the data provides a simple and fast mechanism that should be amenable to hardware optimisation. After a period of relative obscurity, these ideas had a renaissance in the 1970s as *RAM-nets* or *N-Tuple methods*, and particularly in the 1980s where they benefited from some of the mindshare developed by the renewed interest in neural networks under the name of *weightless neural networks* where Austin (1998) collects together some of the more advanced work in this area. There are resonances from those ideas in the work presented here.

Another more contemporary neuromorphic approach using custom system and routing hardware and multiple FPGAs is inspired directly by brain connectivity patterns and provides an alternative set of trade-offs for energy, scaling and speed in realistic neural simulation and learning scenarios (Yang et al., 2021b).

### 2.6. Kernel methods and their mapping into high-dimensional feature spaces

Kernel methods have proven to be a powerful and versatile tool in many areas of machine learning (Shawe-Taylor and Cristianini, 2004; Rasmussen and Williams, 2006). By exploiting only the similarity/difference between cases and projecting (usually non-linearly) into high-dimensional feature spaces that match the data distribution in some sense, both continuous approximation and discrete classification problems can be solved accurately and with few assumptions. The practical limitations are primarily due to the quantity of data and the expensive **O**(*n*^3^) linear computations that usually result, and the necessity of finding closed-form kernel functions for practical efficiencies, in particular for inference.

As discussed more thoroughly in Section 6, we see a number of analogies between Kernel methods and the ideas that we are exploring here. Our method can be seen as constructing a non-linear projection into a high-dimensional feature space and dot products in this space can be used to assess similarity or difference and generate impressive inference accuracies considering that it is a simple and automatic algorithm. Both methods are basically non-parametric, i.e., using the data themselves for inference rather than parameters learned from them. It is also the *coincidences between* our analogue of feature detectors abstracted from synaptic clusters (Mel, 1992) that are at the heart of our method and these too could be seen as dot products in some space. We hope that a further understanding of these parallels will provide a better foundation for the theory of our method.

Neal (1996) elucidates and explores interesting parallels between Kernel methods (specifically *Gaussian Processes*) and neural networks.

## 3. An overview of the basic mechanisms

**Figure 1:**
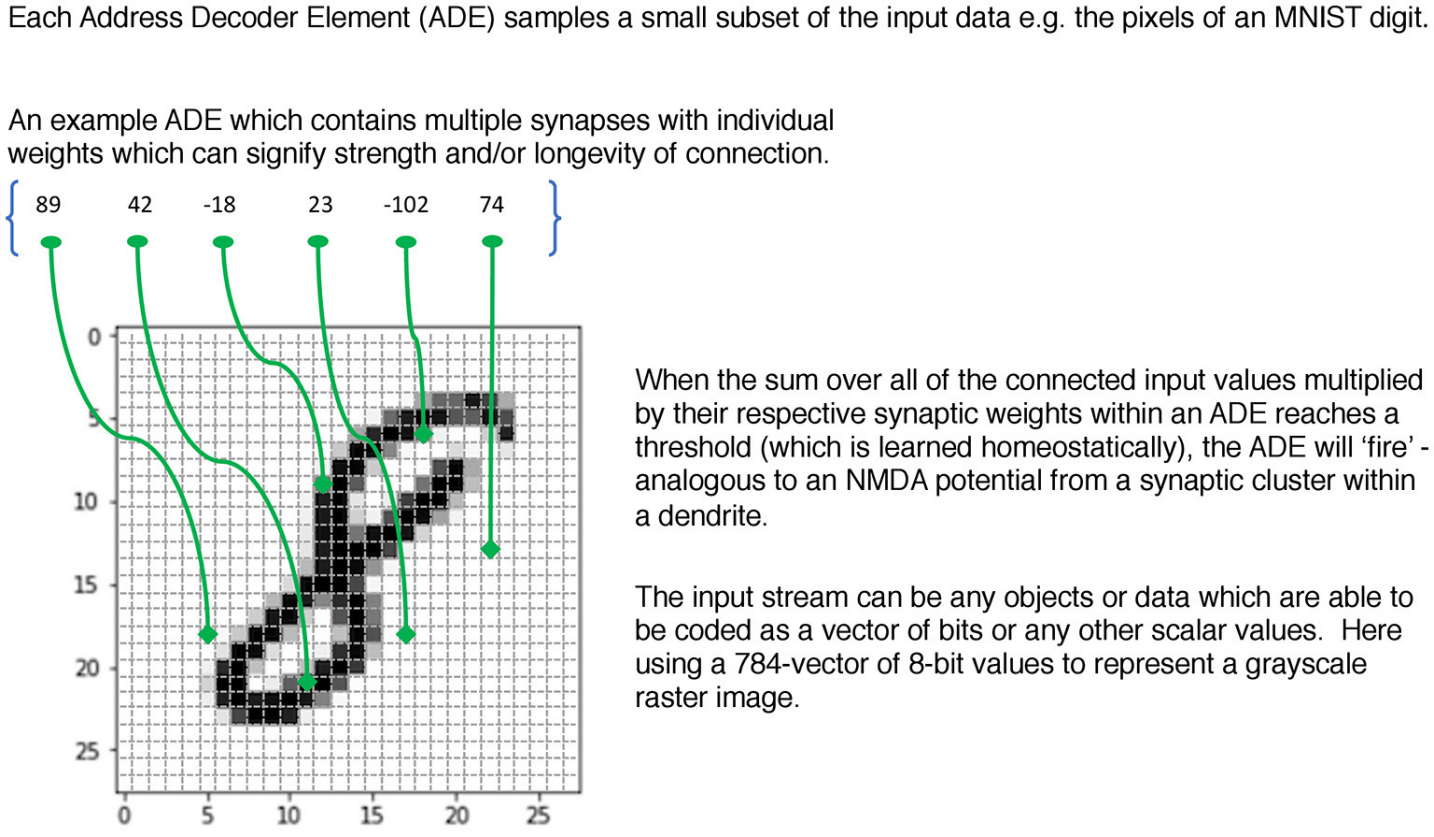
An example ADE subsampling a greyscale raster input from MNIST.

Taking inspiration from the conception of synaptic clusters and their ability to both create and learn from local NMDA plateau potentials, a key concept within the SBC memory is that of an Address Decoder Element (*ADE*). This subsamples from the input stream—initially in a random fashion—and then during the unsupervised learning phase each ADE “homes in” on a feature and at the same time learns a homeostatic threshold θ to facilitate a target firing probability that creates sparse activity patterns. By adding delays of differing values to the synapses in an ADE one can also detect temporal coincidence patterns and so carry out a combined spatio-temporal classification for input data where this is relevant. Figure 1 illustrates the basic mechanism.

In the form of an equation where *j* indexes every ADE.


(1) $$\begin{array}{l} \forall_{j}\;activation_{j}\;=\;\sum_{i=1}^{synapses(ADE_{j})}input_{i}\times weight_{i} \end{array}$$

where *synapses*(*ADE*_j_) = 6 in Figure 1. The homeostatic threshold θ_*j*_ has been learned for each ADE during the unsupervised phase. Then


(2) $$\begin{array}{l} \forall_{j}\text{ if }activation_{j}\geq \theta_{j}\to ADE_{j}\text{ fires} \end{array}$$

The ADEs can be organised in flexible ways. One method that is convenient for software exploration and a simple description is in vectors which we will call Address Decoders (*AD*s) as they now look similar to more conventional memory mechanisms. During the supervised learning phase that follows, the coincidental firing between pairs (or higher-dimensional *n*-tuples) of the ADEs are used to access a memory structure for writing according to certain rules that can be adapted to the particular problem in various useful ways (e.g., choice of class encoding, delays to induce robustness and/or control memory occupancy, biases between classes to improve quality of inference). An equation defines this coincidence mechanism where *j* & *k* are indices over the lengths of each AD


(3) $$\begin{array}{l} \forall_{j,k}\text{ if }AD1_{j}\cap AD2_{k}\to \text{set }SBC_{jkl}\text{ where }l=class \end{array}$$

During inference, the ADEs are driven by test cases and the now populated memory is read using these same coincidence patterns and a simple function of the count of active memory location by class is used to make a class prediction.

**Figure 2:**
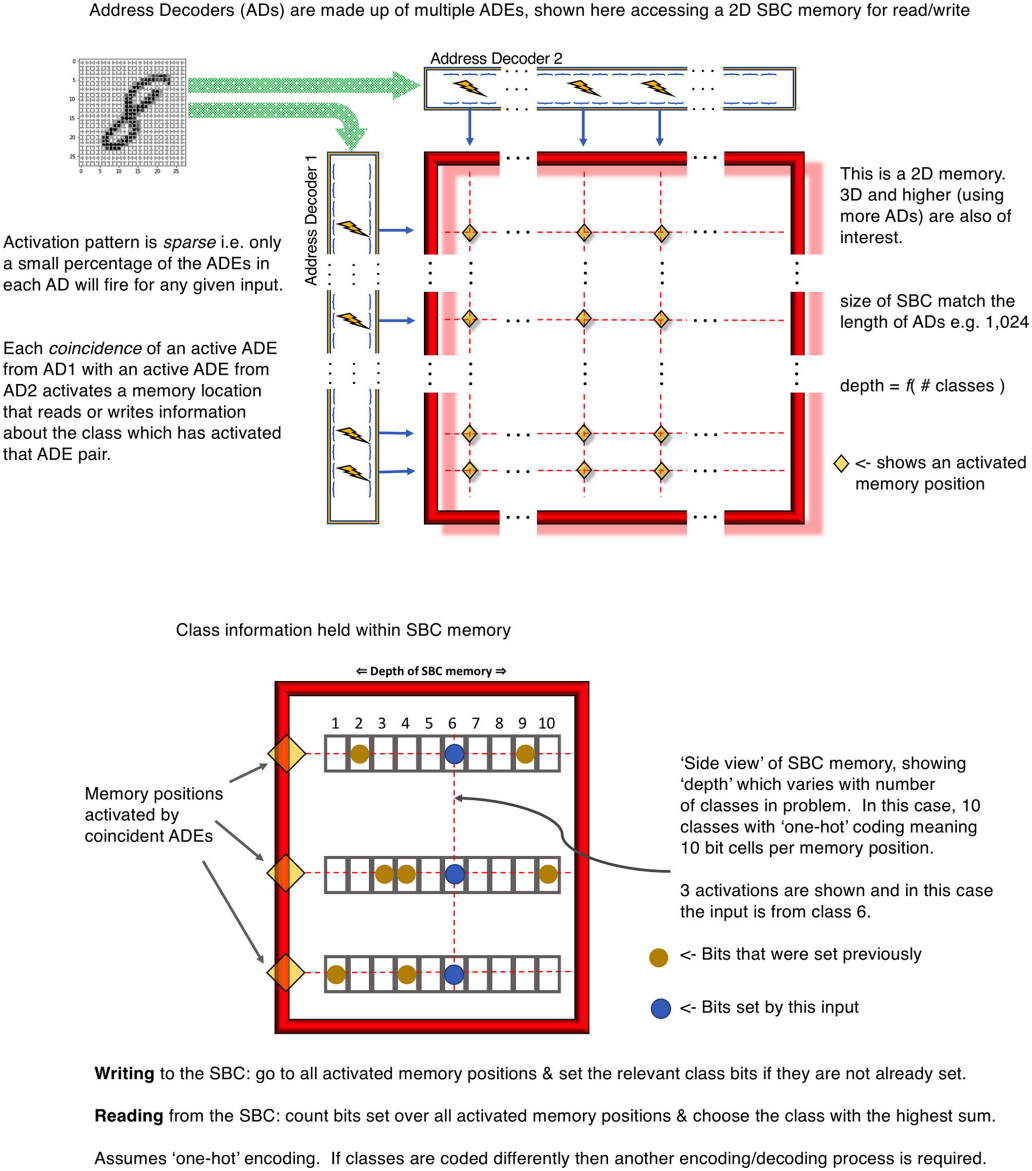
Upper panel is an example 2D SBC memory using 2 ADs. Lower panel is an example class encoding using the “depth” dimension of the 2D SBC memory.

For example, imagine a 2D memory with different ADs along the row and column edges. Each ADE in these ADs connects to a different subset of the input data and has learned a different feature. Typically, the width (i.e., the size of subsample ≡ number of synapses in the cluster) of the ADEs would be different between ADs but the same within an AD. This allows the ADEs within an AD to learn features of similar sizes, whereas those in different ADs learn features of different sizes, perhaps analogous to pooling or convolutional nets of different scales. The upper panel of Figure 2 illustrates this mechanism for a single 2D SBC memory.

Each point formed by the coincidence of 2 ADEs is an accessible region of memory. This region stores a set of the number of classes required in the input data using an encoding appropriate to the problem. The simplest mechanism is “one-hot” encoding. This is illustrated in the lower panel of Figure 2 for 10 classes, each represented by a unique bit in the memory “depth”.

## 4. A sample *BitBrain* implementation

This section gives an overview of the processing steps required for a basic *BitBrain* implementation. This should just be seen as a bare-bones description to clarify what is required.^2^ In Section 7.1, we outline a number of interesting variations that we already have some experience with and there will certainly be other novel developments as the technology matures. To fix ideas, we will assume that the data input is a 784D vector as used in the MNIST and EMNIST examples given in the next section.

### 4.1. Using the global data distribution

Firstly, the global distribution of data over the input vector is calculated over the training set. This is as simple as summing every pixel value into one of 784 bins whilst ensuring no overflow. This vector is then passed to a Metropolis-Hastings (M-H) sampling algorithm [see, for example, Section 5.5 of Bernardo and Smith's book (Bernardo and Smith, 2000) or Section 8.71 of O'Hagan's book (O'Hagan and Kendall, 1994)] which is a reliable method for drawing pseudo-random samples from an arbitrary distribution—in this case generating synapse connections into the input space.

Although simple to achieve, there is no need to normalise this vector to a genuine probability distribution because the M-H algorithm that works with it only requires relative probabilities. In fact, it is not the actual global distribution that is used here but the *sqrt*() of the distribution. There are two possible reasons why this appears to work optimally on problems that have been explored so far:

1. These can be seen as counts and therefore each bin has a Poisson distribution. This means that the uncertainty rises with the mean. The *sqrt*() of a Poisson distribution is approximately homoscedastic above very small counts i.e., the uncertainty becomes independent of the mean.
2. By taking the *sqrt*() of the distribution we are “flattening” it and therefore allowing synapses to be sampled slightly outside of the training data distribution. There are good reasons to believe that this is a good idea for working with data not yet seen in the training set.

It is possible that for data other than greyscale pixels different transformations may be useful, or perhaps a different approach altogether may be preferable at this stage.

### 4.2. Initial AD and ADE setup

We choose *w* for the length of the ADs, let's say *w* = 2,048. This will also define the size of the SBC memories. Larger is typically better but slower and there are diminishing returns beyond a certain point. This is likely to differ between problems. An initial default value for the threshold is set for each ADE. These will be adapted as necessary during the unsupervised learning phase.

Each AD is likely to contain ADEs of the same width *n* (i.e., the number of synapses in the ADE) but typically *n* will differ for each AD. This allows each AD to work with features of different sizes which is useful in image processing and may also be useful with other data types. Each synapse is assigned to one pixel of the image by drawing from the global target distribution—calculated above—using M-H sampling. Hence, pixels which appear more in the data are more likely to be sampled and pixels in the corner with almost no “ink” and no variation between images and classes (and therefore no information) will almost certainly not be sampled. Currently multapses (where more than one synapse per ADE connects to the same pixel) are disallowed.

### 4.3. Choice of synapse types and data

There are several choices available here; we will describe two current ones. One can use binary synapses (i.e., either positive or negative) and their weights then relate to the “longevity” of the synapse which is used during the unsupervised learning phase described below. Initial values of longevity are also set here. Alternatively, one can use 8-bit signed weights drawn pseudo-randomly from some distribution such as uniform or Gaussian which are used to multiply the pixel values.

### 4.4. Preprocessing of the pixel data

In all cases, the pixel greyscale values are centred either side of zero by subtracting 127 from the original 8-bit unsigned values. This provides a bipolar input which can then be multiplied by the weights described above. Early versions of the algorithm discarded all greyscale information by using binary synapses and thresholding the greyscale inputs. The loss in performance was very small and this version is a more natural match to event-based inputs that are becoming increasingly common in neuromorphic sensors.

### 4.5. Clustering of synapses for image and related data

In the case of image or volumetric data it is likely that enforcing a locality constraint will be useful (Dahmen et al., 2022) so that pixels chosen in each ADE must be from the same part of the image. To enforce this, if one draw of M-H sampling does not conform to this constraint then another draw is made until one is found. This can be justified either from a knowledge of the organisation of the retina (Masland, 2012) or by analogy with convolutional front ends in DNNs. This distance may be calculated from any of the other pixels in the ADE which then allows feature detectors of various shapes, or else it can be calculated from a centroid which encourages spherical feature detectors.

### 4.6. Unsupervised learning

To establish a simple form of homeostasis and sparsity as discussed in Section 2 we run through the training set (either in order or, preferably, drawing randomly from it so as to avoid order biases) and, for each training example, establish the ADEs that fire. If drawing randomly we can continue for more than the number of training cases. We accumulate the number of firing events per ADE and after an interval *t* (perhaps 2,000) compare that to the target number of firing events, e.g., 1% of the cases. If it is too high or low we increase or decrease the threshold accordingly. The end point is a threshold which ensures ≈1% firing on average.

During this process we can also carry out a simple Hebbian learning mechanism per synapse within each ADE. One version is related to a simple idea first explored by Hopkins et al. (2018) inspired by NMDA plateau potentials in a synaptic cluster. If the ADE fires then the smallest contributor to the sum which led to the threshold crossing has its longevity decremented by 1 and the largest contributor to the sum has its longevity increased by 1. After an interval *t*, if any single synapse has a longevity below a critical value it is replaced using the same mechanism as was used in the original setup of the ADE (as described above); a new pixel is chosen and its longevity is reset to the default value. This allows each ADE to home in on a feature. Neither this nor the threshold learning requires class information, hence this phase is “unsupervised”.

### 4.7. Supervised learning

Now that we have all the ADs setup we can carry out the supervised learning using class information. First there is the choice of SBC architecture. In all cases 2D SBCs with one-hot class encoding are currently assumed. The number of SBCs can be chosen, each using either a pair of distinct ADs or using the same AD for both its row and its column decoder. For example, with 4 ADs each of different width *n*_i_ there are (42) = 6 SBCs where the AD on each row and column is a distinct combination. These SBCs recognise coincidences between ADEs of different widths, where the feature sizes differ. In addition, there are 4 possible SBCs that recognise coincidences between ADEs of the same width. These are half-size SBCs because only one half of the off-diagonal elements of the SBC are describing unique coincidences. An intelligent implementation will fit two of these half-size SBCs into the storage for one full-size SBC. So for example, the first 6 will be AD_1_ * AD_2_, AD_1_ * AD_3_, AD_1_ * AD_4_, AD_2_ * AD_3_, ... , and the last 4 will be AD_1_ * AD_1_, AD_2_ * AD_2_ etc. Other good results have been obtained using a simpler setup: 3x ADs with (6, 10, 12) synapses placed randomly at (+/−2, +/−3, +/−4) in *x* and *y* relative to a centroid chosen by M-H sampling. 3 SBCs are used, each using a different pair of the 3 ADs with no half-size SBCs.

The SBCs are now populated using a simple single-pass supervised learning mechanism that lies at the heart of the method. In a single pass through the training data all coincidences between ADEs cause the respective class bit to be set in the SBCs. A specific class bit may be set many times by different training examples, with the same outcome as if it were set only once by one training example. After a single pass through the training set, supervised learning is complete. An additional pass would, in any case, have no additional effect on the SBC contents unless training noise was being added to increase robustness as described in the next section though the differences are likely to be very small in realistic cases.

### 4.8. Inference

The inference and supervised learning mechanisms are very similar, accessing the relevant SBC locations in exactly the same way, setting respective class bits during training but instead counting the set bits during inference. For inference an input case is acquired from a test set and presented to the ADs. For each coincidence between ADEs all class bits are read from the corresponding location in the relevant SBC, and the number of bits set for each class is summed across all SBCs. The highest sum indicates the inferred class of this test case.

If the classes are not one-hot encoded then there is an extra decoding phase required here to identify the most likely class from the accumulated bit counts.

## 5. Example results using MNIST and EMNIST

Some results are given below for two standard classification problems, the first very well-known and relatively easy, the second less so. They both provide input data as greyscale raster plots of handwritten digits/characters and require the correct classification over a test set once the training set has been digested by the learning mechanism.

In both of these cases, the basic *BitBrain* algorithm with one-hot class encoding, as described in the previous section, has been used. Improvements are possible using spatial jitter at the training stage, a technique termed *data augmentation* in the machine learning literature, but for simplicity and clarity we present raw results here. The test setup for these results is 4 ADs with different ADE widths {6, 8, 10, 12} where the subsampling pattern for each ADE is spatially clustered. The ADs each contain 2,048 ADEs and there are 10 2D SBC memories; 6 of which are full-size 2-way coincidences between different ADs, the other 4 being half-size memories containing coincidences within one AD as described above.

In each case we present results for varying amounts (including zero) of noise added independently per pixel during the training and/or testing phases. This noise can take one of two forms: Gaussian noise of the specified SD with maximum and minimum clamped at 255 and 0 respectively, and “Salt and Pepper” noise with a given probability of a pixel being replaced with 0 or 255, with each of these values being equally likely. Zero noise for both training and test is comparable to standard results. Noise added to the test set simulates imperfect inputs. Noise added to the training set helps to make inference more robust to test noise as can be seen in the plots.

For *BitBrain*, the uncertainty due to different random number seeds can be assumed at ≈0.1% on the Y axes which is too small to be represented by error bars so the thickness of the lines is a good guide. This represents another form of robustness for the learning process itself.

### 5.1. MNIST

**Figure 3:**
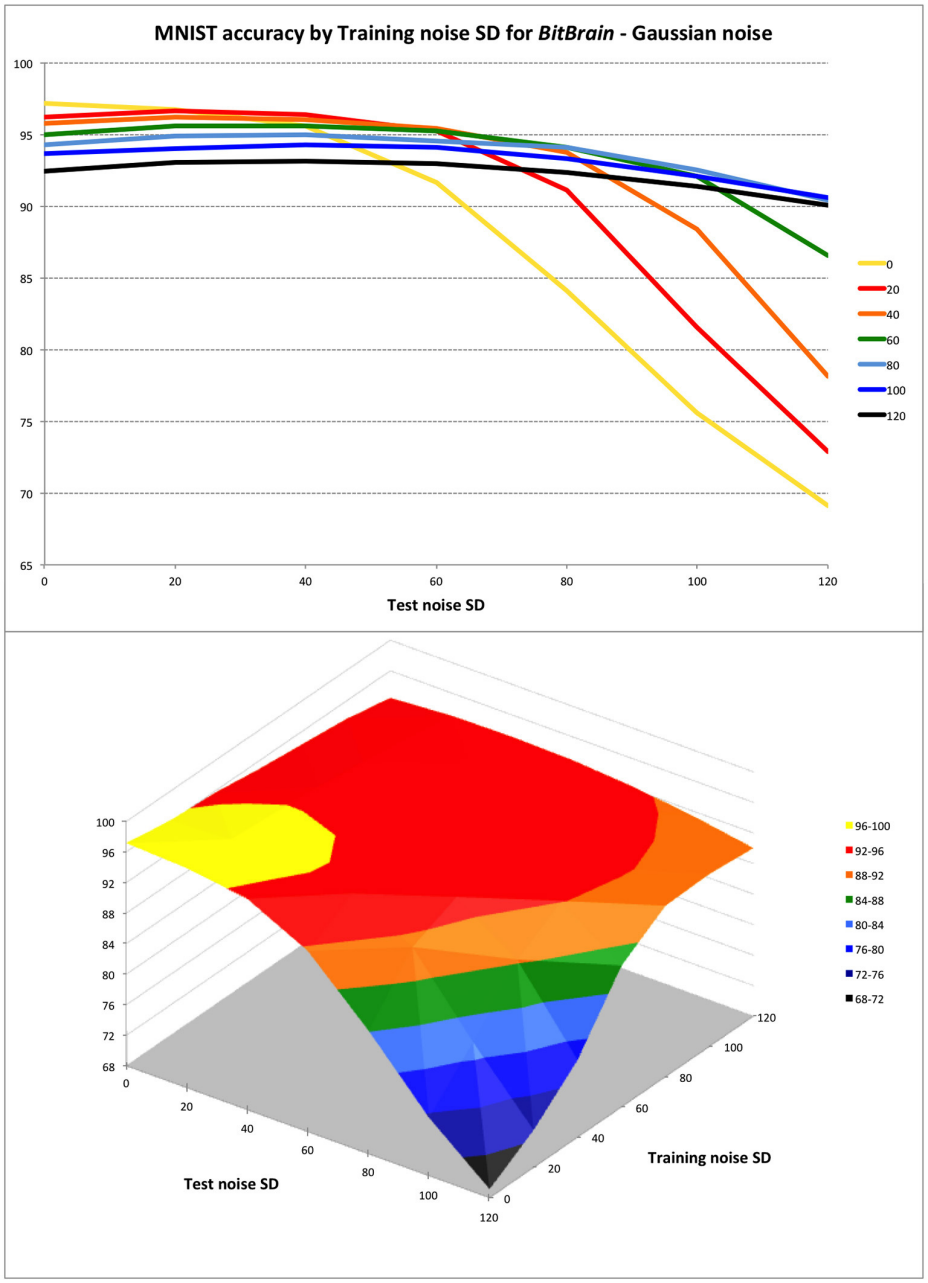
*BitBrain* performance against training and test Gaussian noise levels. Training noise SDs indicated in the legend for top plot and on second axis in surface plot. The trade-off between inference performance with perfect and noisy test data is clear. To perform well with inputs that are very noisy or otherwise imperfect, a small penalty must be accepted with perfect input data by training with appropriate amounts of noise. Over this range of test noise a training noise of 40–60 SD seems to be a good compromise; not penalising performance badly with perfect data whilst protecting against degradation with quite large quantities of test noise.

This is the standard MNIST problem and data set (Deng, 2012) using 60,000 training images labelled with the 10 digit classes and 10,000 test images. In Figure 3, we give two views of the robustness performance of the setup described. In order to optimise expected performance in a real-world application a view must therefore be taken on the quality of the input data likely to be encountered. Perfect input data is unlikely in any realistic scenario (unlike benchmark testing), and this graceful degradation in real-world usage is one of the primary drivers for our interest in these ideas.

To give some idea of inference speed for this problem, an implementation of *BitBrain* was set up with 3 ADs each containing 2,048 ADEs driving 3 full-size 2D SBC memories, requiring 16 MB of memory for the total SBC footprint. Running on a 2020 MacBook Air laptop with a 3.2 GHz Apple Silicon CPU this took around 7 s for supervised training on 60,000 examples and 0.42 s for the 10,000 test inferences, delivering 96.6% accuracy with no training or test noise. This was single-threaded C code on the default compiler with no attempt to optimise beyond good coding practice, and no use was made of the GPU.

It is instructive to compare the robustness performance against some representative CNNs which represent a technology designed expressly for such image classification tasks. *LeNet-5* was an early breakthrough and reference designed for handwritten digit recognition (LeCun et al., 1998) which performs to a similar standard to our default *BitBrain* setup in the presence of noise-free inputs. *Efficient CapsNet* (Mazzia et al., 2021) is very recent and arguably close to state-of-the-art, so therefore a very challenging comparison.

*Efficient CapsNet* models were trained for a maximum of 100 epochs with ReLU activations, while the training setup for *LeNet-5* was a maximum of 100 epochs and sigmoidal activations. To save computational effort we used Tensorflow and some simplifications to the weight setup and training schedule. Also, there is no canonical Tensorflow implementation and the original *LeNet-5* paper uses a 32 × 32 version of the MNIST data. Together these are reasons why our noise-free results are not quite as good as the original results, but perhaps more importantly in this context they are comparable to *BitBrain* on noise-free data. Despite these small differences, we are confident that the important trends over training and test noise values will be unaffected.

In all cases we apply the same noise pattern per training image which is then frozen over training epochs. We call this *static* noise and the aim is to try and provide a fair comparison with *BitBrain* because during our key supervised learning phase each image is (in this study) only seen once and therefore contaminated with only one realisation of the noise distribution. This is not necessarily the case during our unsupervised learning phase, however we believe this is a secondary consideration. In any case, during a small number of test runs we have found that *LeNet-5* results were not significantly improved using *dynamic* noise where a different noise pattern per image is produced for every training epoch.

**Figure 4:**
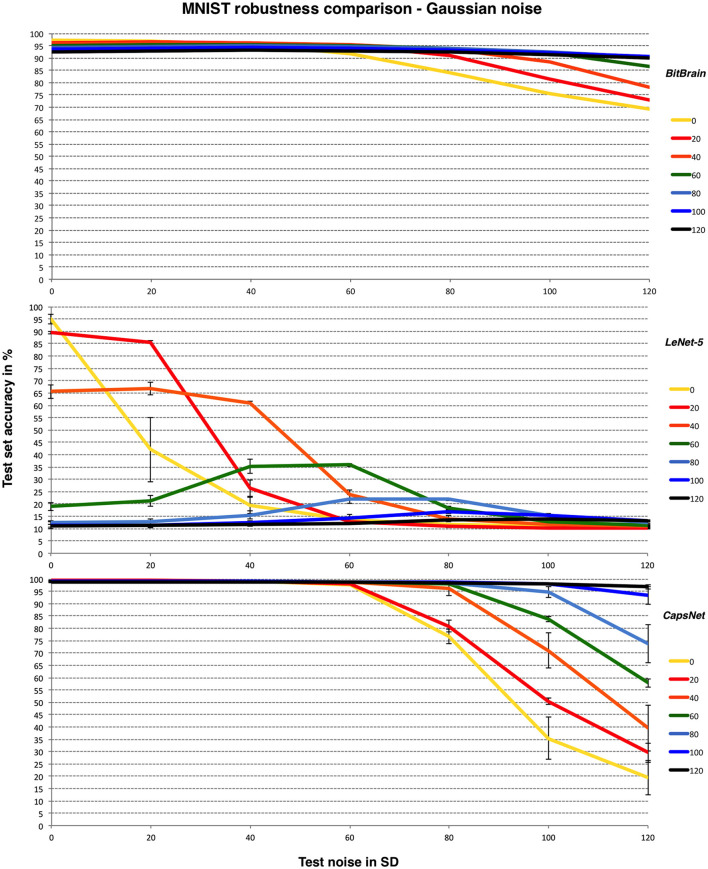
*BitBrain* **(top)** vs. *LeNet-5* **(middle)** vs. *Efficient CapsNet* **(bottom)** robustness comparison for MNIST and Gaussian noise with bounded pixels. Training noise SDs are the lines identified in the legend, test noise SD is on the X axis. *LeNet-5* results are mean and SD error bars from 8 independent runs. *CapsNet* results are mean and SD error bars from 3 independent runs.

For the *LeNet-5* results there is considerable variation in the accuracy results with different random number seeds. We believe this is the combined effect of differing startup configurations and noise distributions over the training images. As a result, we have shown mean and SD error bars for these results from a small number of independent runs. It is worth noting that this sensitivity to setup conditions is another form of non-robust behaviours independent of the one that we are aiming to test here, but with its own practical implications. For *Efficient CapsNet* we have limited runs available due to time constraints; indicative error bars are given but these are less precise than those for *LeNet-5*. Figure 4 compares *BitBrain* and the two CNNs on the same Y-axis with the MNIST data set and Gaussian noise.

Clearly *LeNet-5* suffers badly in the presence of noise here but with an interesting pattern of the best test noise performance matching the same training noise setting, as if it has learned to recognise the appropriate signal-to-noise ratio. This pattern is even clearer in the middle panel of **Figure 6** and has also been observed in independent work using a different CNN and where modifications of the training and test sets have been distortions other than noise (Adithya, 2022). This is suggestive of another kind of overfitting where the CNN is only learning to recognise data with one particular signal-to-noise ratio or contrast level, and is therefore lacking inferential robustness in realistic real world scenarios. It may be that this is a fruitful area of investigation for future study. *CapsNet* is far more robust and in fact responds very well to high values of training noise. Presumably this acts as an effective regulariser which may be an interesting discovery. *BitBrain* is the least affected by different amounts of training noise at higher test noise levels but does not quite reach the accuracy levels of *CapsNet*.

**Figure 5:**
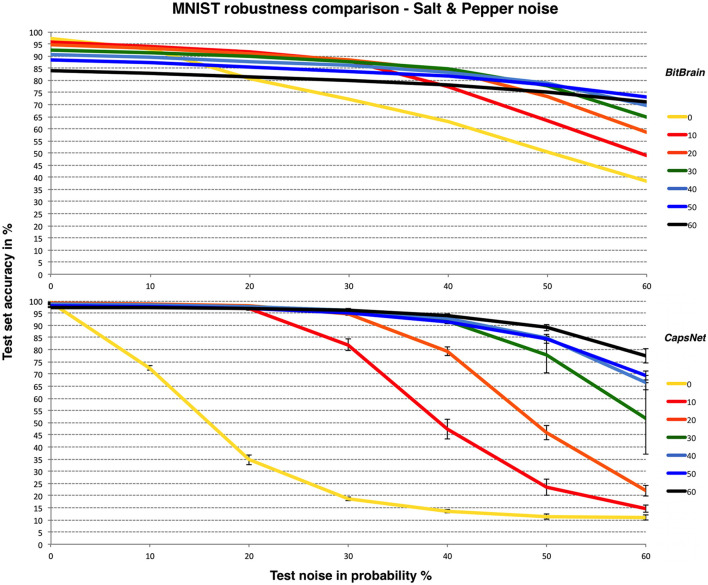
*BitBrain* **(top)** vs. *Efficient CapsNet* **(bottom)** robustness comparison for MNIST and Salt & Pepper noise. Training noise probabilities are the lines identified in the legend, test noise probability is on the X axis. *CapsNet* results are mean and SD error bars from 3 independent runs.

We thought it would be interesting to show a similar comparison between *BitBrain* and *CapsNet* for a different type of noise: “Salt and Pepper” as described at the start of this section. *LeNet-5* appears unable to produce consistent results with this form of noise over these ranges. This is shown in Figure 5.

### 5.2. EMNIST

EMNIST (Cohen et al., 2017) is a problem similar in nature to MNIST (i.e., 28 × 28 raster plots of greyscale digitised handwritten characters) but much more challenging. All digits and lower- and upper-case characters are used in the most comprehensive *by_Class* data set where the 62 classes are significantly unbalanced and several characters effectively alias each other, e.g.,

{o, O, 0}, {i, I, l, 1}, {s, S, 5}, {B, 8}

**Figure 6:**
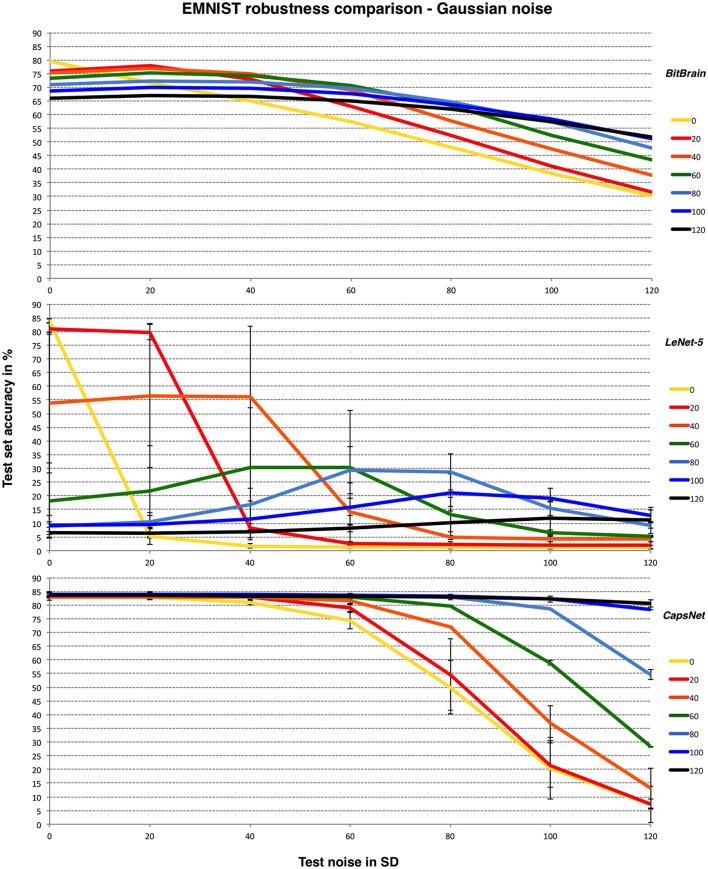
*BitBrain* **(top)** vs. *LeNet-5* **(middle)** vs. *Efficient CapsNet* **(bottom)** robustness comparison for EMNIST and Gaussian noise with bounded pixels. Training noise SDs are the lines identified in the legend, test noise SD is on the X axis. *LeNet-5* results are mean and SD error bars from 6 independent runs. *CapsNet* results are mean and SD error bars from 3 independent runs.

Figure 6 provides the results along with the CNNs as in the previous subsection.

Here we achieve results significantly better than the original results for noise-free operation (Cohen et al., 2017) with our basic mechanism, though more recent work has moved the achievable bound upwards by a few percent over ours (Baldominos et al., 2019) as can be seen from the CNN results here. The trade-off between noisy and noise-free test data here is clearer and accuracy generally much lower due to the nature of the problem. Again, adding an appropriate amount of training noise protects the initial performance effectively across a wide range of test noise though with a greater penalty for noise-free data.

**Figure 7:**
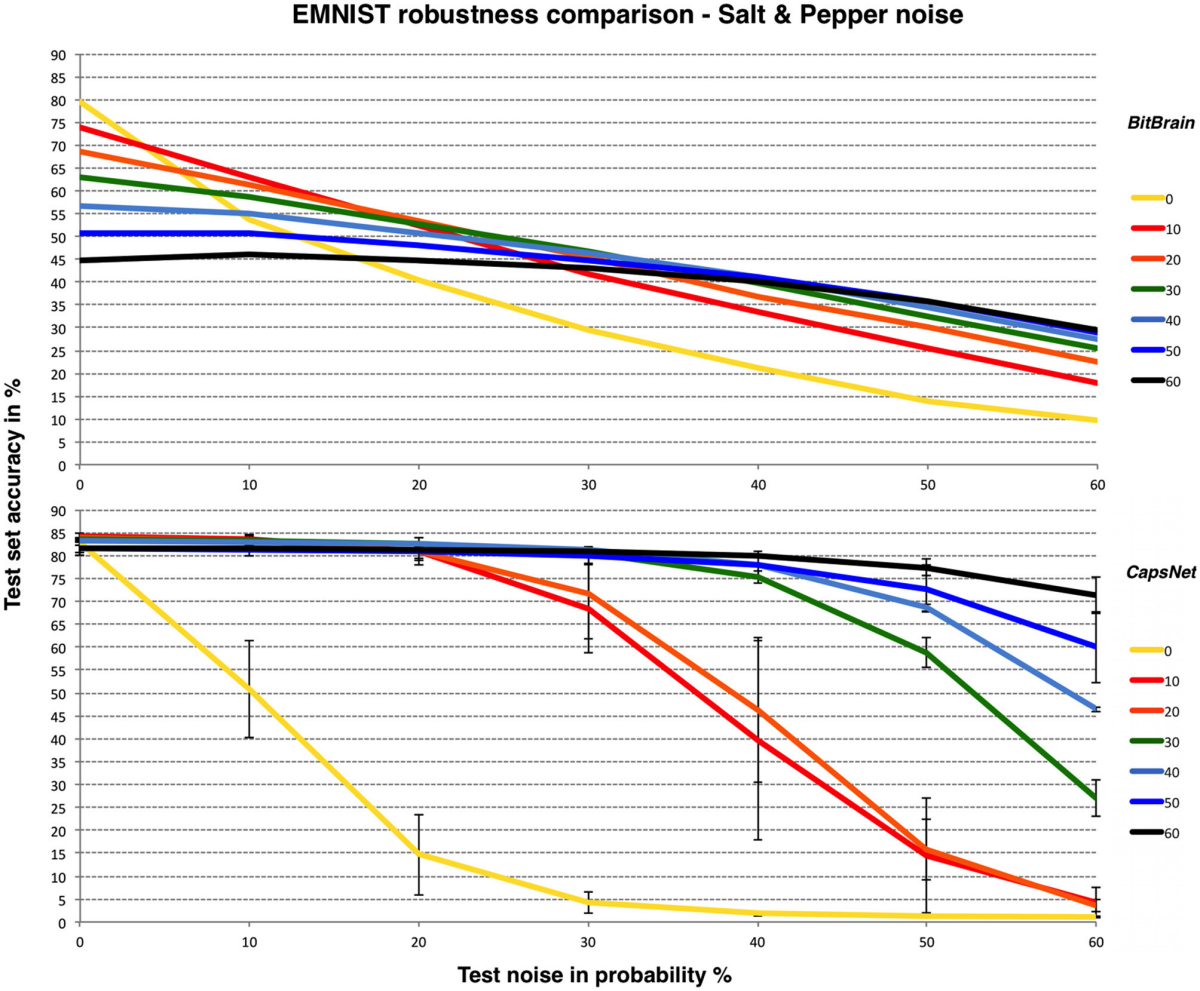
*BitBrain* **(top)** vs. *Efficient CapsNet* **(bottom)** robustness comparison for EMNIST and Salt & Pepper noise. Training noise probabilities are the lines identified in the legend, test noise probability is on the X axis. *CapsNet* results are mean and SD error bars from 3 independent runs.

Similar patterns are observed here. *LeNet-5* suffers most and has very high variability, *BitBrain* is least affected by different training noise settings at high test noise and *CapsNet* again performs very well with high training noise. Despite being completely unrelated technologies, both *BitBrain* and *CapsNet* respond well to high levels of training noise. We again compare *BitBrain* and *CapsNet* using Salt and Pepper noise in Figure 7.

The EMNIST problem combined with Salt & Pepper noise at these levels is obviously a significant challenge for both technologies, though again *CapsNet* with high levels of training noise performs very well.

### 5.3. Comparison with other single-pass ML methods

In this section, we present a summary of results from the ML literature about single-pass learning, where each sample from the training set is used only once and is not stored in memory. We investigate how *BitBrain* in its current form compares to a number of natively single-pass approaches (Wang et al., 2012, 2013; Zhou et al., 2016), well-established deep neural networks (He et al., 2016; Mazzia et al., 2021), and two simple CNNs with one and two convolutional layers trained with just a single epoch.^3^ We continue to use MNIST here as the results are widely reported.

Although the state-of-the-art modern machine learning models often rely on deep networks which are successively trained over many epochs, simpler approaches which need only a single pass through the training set are still of interest to the community, especially in applications with limited resources. These single-pass approaches typically employ a form of online learning which allows them to process large datasets without the need for excessive computational resources. One particular approach—local online learning (LOL) (Zhou et al., 2016), proposes an extension of commonly used Passive-Aggresive (PA) method (Crammer et al., 2006) which updates the classifier sequentially based on the feedback from each data point in the training set. Unlike the PA and related approaches, the LOL allows for learning multiple local hyperplanes to non-linearly process sequential data in a one-pass manner. The authors also introduced a novel optimisation strategy which significantly improves the performance on classification tasks with multiple classes of patterns compared to previously proposed methods.

**Figure 8:**
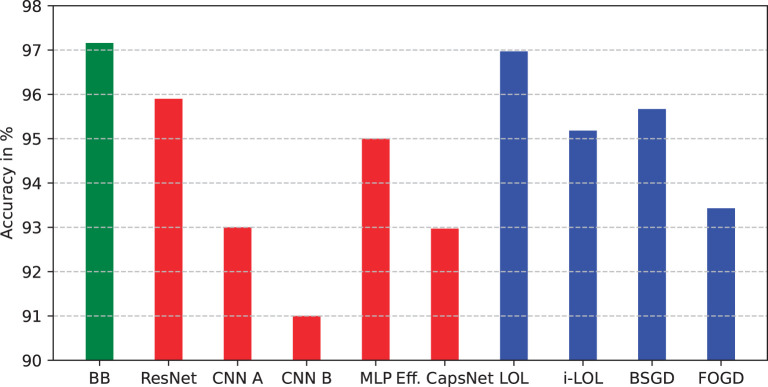
Performance comparison between *BitBrain* (green bar), deep neural networks (red bars), and natively single-pass learning approaches (blue bars). Accuracy in % on the test set.

Figure 8 shows the single-pass performance of deep learning methods (red bars) and natively single-pass methods (blue bars) in comparison to *BitBrain* (green bar). The performance of *BitBrain* is visibly better than deep learning methods, which significantly underperform when trained with just a single epoch, as well as the online single-pass approaches. Notably, natively single-pass approaches also provide a better classification accuracy than more commonly used convolutional neural networks, however this discrepancy is likely to result from the fact that the training hyperparameters of the CNNs have not been adjusted adequately in such a limited training time.

Another single-pass comparison can be made with online methods for SVMs in two-class problems (Rai et al., 2009). In Table 1, results are provided from MNIST for discriminating *0* vs. *1* and *8* vs. *9* using a number of different algorithms.

**Table 1:** Two-class results for accuracy in % compared with *BitBrain* from Table 1 of Rai et al. (2009).

**Task**	**libSVM**	**Perceptron**	**Pegasos1**	**Pegasos20**	**LASVM**	**StreamSVM1**	**StreamSVM2**	***BitBrain***
*0* vs. *1*	99.52	99.47	95.06	99.48	98.82	99.34	99.71	99.95
*8* vs. *9*	96.57	95.90	69.41	90.62	90.32	84.75	94.70	98.49

The relevant parts of their table are replicated here with the best result for each case in red.

### 5.4. Single-shot performance

**Figure 9:**
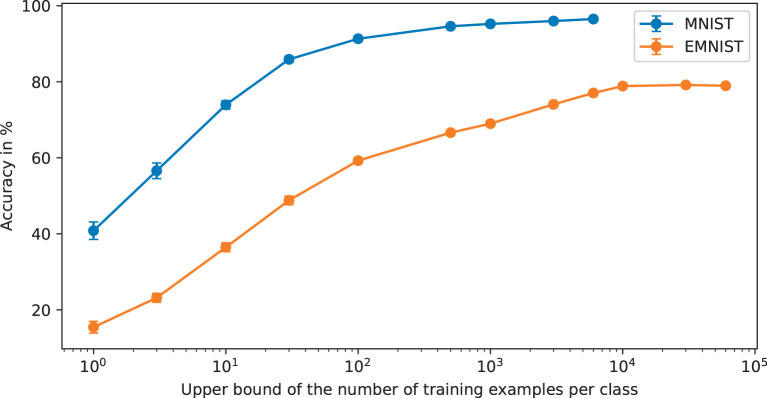
Accuracy as a function of the upper bound of the number of per class training examples. The X axis is less obvious for EMNIST because as we move further to the right some of the under-represented classes will have their training sets exhausted whilst other classes are still being subsampled. It may not be obvious but beyond 10,000 training examples on EMNIST the accuracy falls very slightly because the over-represented classes are still being added into the SBCs when there are none of the under-represented class training examples left which exacerbates the unbalanced nature of the data set.

In this section, we present some results that show learning performance as a function of *n* for MNIST and EMNIST with very small training sets from *n* = 1 per class upwards. These are shown in Figure 9 where error bars are one standard deviation from 10 repeats with different randomly generated subsets of the training cases. These results show that training data sets far smaller than are common in current machine learning applications can be useful in terms of generating inference accuracy well beyond chance. This will have implications for where *BitBrain* can be applied. It is also worth noting that the error bars are very small, even for *n* = 1. This indicates another aspect of robustness demonstrated by the *BitBrain* mechanism because it hardly seems to matter which training cases are chosen and anyone familiar with the MNIST and EMNIST data sets will know that the training cases can vary substantially.

## 6. Relationships to Kernel methods theory

*BitBrain* is a new idea and the underlying theory has to be developed further in order to catch up with the empirical results. This will help guide future directions for research and improve practical results and implementations. In Section 2 of the main document we discuss ideas from a number of fields which have informed this technology. In this section, we want to explore one of them further.

Kernel methods are based upon a matrix which is created by the similarities between data points in the training set. This is called the *Gram* or *Kernel* matrix which we will call ***K***. If there are *n* training data this matrix will be *n* x *n* positive definite symmetric (PDS) with “self-similarities” (however that is defined) along the diagonal and all the off-diagonal entries being the similarities between different training data cases.

Similarities can be defined in many ways, the primary constraint being that they must generate a PDS Kernel matrix. A standard description in the kernel methods literature is that the elements of ***K*** are formed by dot products between features so that ***K***_ij_ = < ϕ(*x*_i_), ϕ(*x*_j_) > where ϕ() is an arbitrary function that maps the input data *x* into a corresponding feature space. The choice of ϕ() is therefore key in order to make any given method appropriate for the data involved.

The “kernel trick” which provides potentially very large computational benefits for kernel methods is to find a closed-form function *k*(*x*_i_, *x*_j_) = < ϕ(*x*_i_), ϕ(*x*_j_) > without having to calculate the (perhaps very high-dimensional) dot products required explicitly. A good example from Gaussian Process methods is the covariance function between two points in input space which can be of a very simple closed form whilst at the same time (i) guaranteeing a PDS Kernel matrix and (ii) expressing a very high-dimensional feature space that can be parameterised and adapted easily but which never needs to be explicitly calculated (Rasmussen and Williams, 2006).

### 6.1. A simple multi-class Kernel-based classification (KBC) method

Probably the most straightforward KBC method that can be applied effectively to problems with multiple classes is called *Least-squares Classification* (LSC) (Rasmussen and Williams, 2006) and various versions are compared by Rifkin and Klautau (2004). In its simplest form, assume that ***K*** is formed from the training data and that there are *c* = 10 classes (e.g., for the 10 MNIST digits) with each training case labelled with one of the set {0, 1, ... 8, 9}. Now make *c* “dummy targets” ***y***_0_-***y***_9_ which are of length *n* and in each case contain a zero for training cases where the label doesn't match their subscript and a one where it does. So in this case there are about 90% zeroes and 10% ones for each ***y***_i_. Now generate 10 “hat” vectors ***h***_0_-***h***_9_ of length *n* which are essentially weights (both positive and negative) used for assessing any new case and which class it corresponds to. The algebra^4^ of this is:


(4) $$\begin{array}{l} h_{i}=K^{-1}y_{i} \end{array}$$

So now the class of a new data case can be inferred. For each case form the vector k_*_ of length *n* which gives the similarity (exactly as defined when ***K*** was formed between training cases) between the new case and all *n* training cases. This is like forming a new row/column of ***K***. Now create the dot product of k_*_ with each ***h***_i_ to produce *c “*class indicator” values, i.e.,


(5) $$\begin{array}{l} class\;indicator_{i}=k_{*}.h_{i} \end{array}$$

In the ideal case this would produce *c*-1 class indicators = 0 and 1 class indicator = 1, with the index of the latter providing the class inference. In reality, imprecision in the similarity metric, noise in the training and test data and other issues will make these results approximate, but a simple and robust mechanism for class choice is to pick the largest value. This intuitively describes “overall similarity to training cases of class_i_” in the similarity metric defined by the kernel function chosen.

### 6.2. How does *BitBrain* resemble and differ from KBC methods?

Now think about all possible 2D coincidences from a data input as one long bit vector. For example, assume there are 3 ADs each of length *w* = 2,048 and 3 2D SBC memories to capture all the full-size coincidences. “Unrolling” the 2D bit positions from this setup would make one vector, ***b***, of length 2,048^2^ x 3 = 12,582,912 bits. This ignores the class bit depth, which will be addressed later. The number of possible bits turned on in ***b*** ranges from 0 to 12,582,912. Assume a 1% firing probability per AD and a random distribution for the active bits, then the expected number of bits turned on ≈ 20.48^2^ x 3 = 1,258.3.

Now consider two different inputs. These will turn on different sets of bits in ***b***. Taking the logical AND of these two vectors is also, of course, a dot product, which can then be written in exactly the form < ϕ(*x*_i_), ϕ(*x*_j_) > as described at the start of Section 6. This can be thought of as an overlap between bit vectors or an intersection between bit sets. As it can be expressed as a dot product it is a valid similarity measure which will produce a PDS Kernel matrix, with ϕ() here being a projection of the image data *via* the ADEs into a sparse 12,582,912D binary space

**Figure 10:**
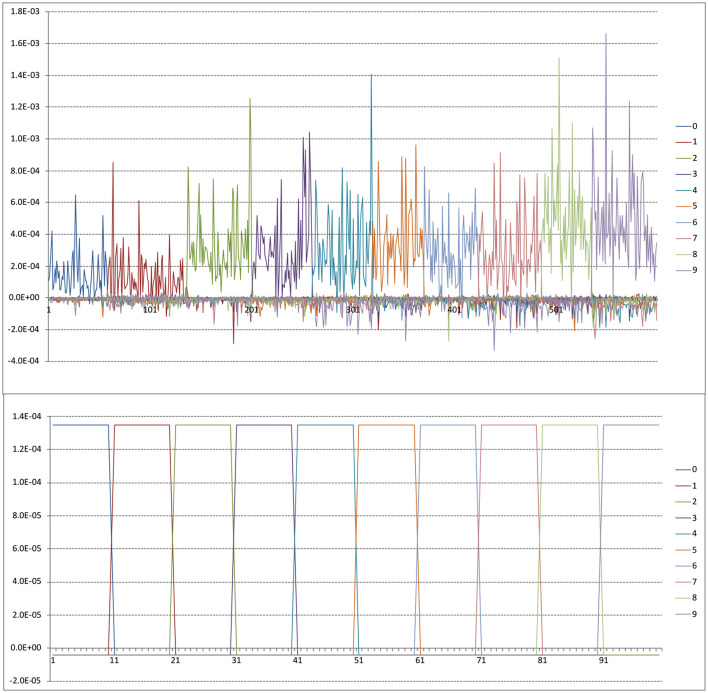
Upper panel is sample ***h***_i_ vectors for first 600 MNIST training cases. Lower panel is synthetic ***h***_i_ vectors for *n* = 100 as described in text.

It may be instructive to try and demystify the ***h***_i_ vectors somewhat. The upper panel of Figure 10 is a plot from the first 600 MNIST cases, sorted by label so that the first ones are 0s, then 1s, etc. As expected by the form of the ***y***_i_ vectors, these ‘gate' the weightings for their own labels. However, there are subtleties as well such as substantial differences between same label cases (caused by the interrelated relative similarities encoded in ***K***^-1^) and some less trivial negative weights as in case 183, which presumably means that case (which is a **2)** is particularly dissimilar to a/some **1** case(s), hence the negative weight.

Using the above setup and the mechanisms described in Section 4.6 we achieved 98.6% on MNIST, which is the best result so far using these ADEs. Table 2 shows all the class indicator values for two correctly assigned sample outputs. The first is obviously a clear result with good confidence and the second less so.

**Table 2:** The class indicator values for two sample outputs.

**Digit**	**ID 3357 is a 5**	**ID 5680 is a 3**
0	0.008482	0.113866
1	0.099251	0.008050
2	−0.003518	0.023137
3	−0.058747	0.414228
4	−0.058338	−0.005619
5	1.087230	0.063122
6	−0.040066	0.010855
7	−0.063182	0.029908
8	0.058970	−0.026898
9	0.039974	0.019438

### 6.3. The relationship to *BitBrain*

In *BitBrain* the same vector ***b*** is formed for every data case but used in a different way. For every training image the class label is stored for every active bit position of ***b*** where it is not already set. This assumes that ***b*** now has a 2^nd^ depth dimension of length *c* (for one-hot encoding), or alternatively think of *c* vectors ***b***_i_ which are analogous in some sense to the KBC “hat” vectors ***h***_i_. The second perspective corresponds more directly to the kernel definitions and is used in what follows.

The essence of understanding the relationship between these two apparently distinct methods is to see how the supervised learning memory write mechanism relates to Equation (4), and how the read operation for inference relates to Equation (5). They are clearly performing related tasks, albeit in very different ways. A current hypothesis is that the *BitBrain* mechanism is forming an empirical approximation to the kernel function *k*(*x*_i_, *x*_j_) described earlier, so that the expensive **O**(*n*^2^) and **O**(*n*^3^) operations required in Equations (4) and (5) are now converted into memory writes and reads over the training data as a whole.

What is not yet clear is exactly how that relationship can be derived, but perhaps some progress can be made. A starting point is the observation that *Mercer's Theorem* means a kernel function can be appropriately decomposed into a summation over products of orthonormal functions, i.e.,


(6) $$\begin{array}{l} \kappa \left(x,z\right)=\overset{m}{\underset{j=1}{\sum }}\phi_{j}\left(x\right)\phi_{j}\left(z\right) \end{array}$$

So that, for example, the ϕ_j_() could be eigenvectors of the ϕ() function that defines the feature space. A suggestion is that *x* is a vector of binary values, *m* = *length*(*b*) and ϕ_*j*_(*x*) = *x*[*j*] where [ ] indexes into the vector and therefore returns one of {0, 1}. Kernel functions such as this are described by Shawe-Taylor and Cristianini (Shawe-Taylor and Cristianini, 2004) in Sections 9.5 and 9.7 as set kernels and by Odone et al. (2005) as histogram intersection kernels which turn out to have a natural link to L^1^ distance in their Equation (15). Their use in image processing problems is described by Raginsky and Lazebnik (2009). So it is possible that the *c* vectors ***b***_i_ are related to this summation, where the summation index is now over their length. As required above, each bit of ***b***_i_ would by definition be (approximately) orthonormal in a large, sparse binary vector.

### 6.4. Differences from Kernel methods

Unfortunately, we don't have direct access to the full Kernel matrix ***K*** which describes the similarities between all training cases and so cannot explicitly form Equations (4) and (5) which are essential for LSC. However, we have generated a union over all the bit patterns found from the training set and stored them in the relevant ***b***_i_ according to their class information. It seems that this sampling and storage mechanism has taken the place of Equation (4) and then Equation (5) is being approximated by the overlap of the bit pattern from a new data input which we can call o_*_ (analogous to k_*_) and the stored bit pattern in each ***b***_i_ (analogous to ***h***_i_).

So what can we say about this? For one thing, in the kernel method the similarities are unambiguously calculated between cases. In *BitBrain* the similarities are calculated between o_*_ and either the bit pattern projections over *length*(***b***) or the *c* classes, depending on your perspective. It may be useful to think about this in terms of set theory. The cardinality of the whole bit set = *length*(***b***). The subsets for each class are defined by the active bits in each ***b***_i_ let's define these subsets as **S**{***b***_i_}. The relationship between any new point and the stored training cases for class_i_ is defined by the intersection of the active bits in o_*_ with those of **S**{***b***_i_}. We can directly call this a similarity by appealing to Equation (6). This gives us half of Equation (5) but where do we get the equivalent of ***h***_i_ and how do we deal with the different domain over which the dot product is calculated?

To address this question it might be useful to consider the structure of the ***h***_i_. If in the kernel setup all cases are equally similar to cases in their own class, and equally but less similar to cases in other classes then ***h***_i_ just looks like a gating variable. A synthetic simulation for *n* = 100 is shown in the lower panel of Figure 10, sorted by class. This was created by a ***K*** matrix with 3,000 on the diagonal (i.e., self-similarity or the number of bits activated by a case), 500 off diagonal for cases of the same class and 50 off diagonal for cases of different classes.

It's interesting to compare this with the upper panel of Figure 10 where varying mean similarities by class and within-class similarities between specific cases produce a very complex pattern of weightings. In this case the exact (and unrealistic) uniformity of similarity provides a very clear weighting pattern. Arguably, what we are doing with *BitBrain* is the same as this but gated across *c* classes or *length*(***b***) depending on your perspective.

**Figure 11:**
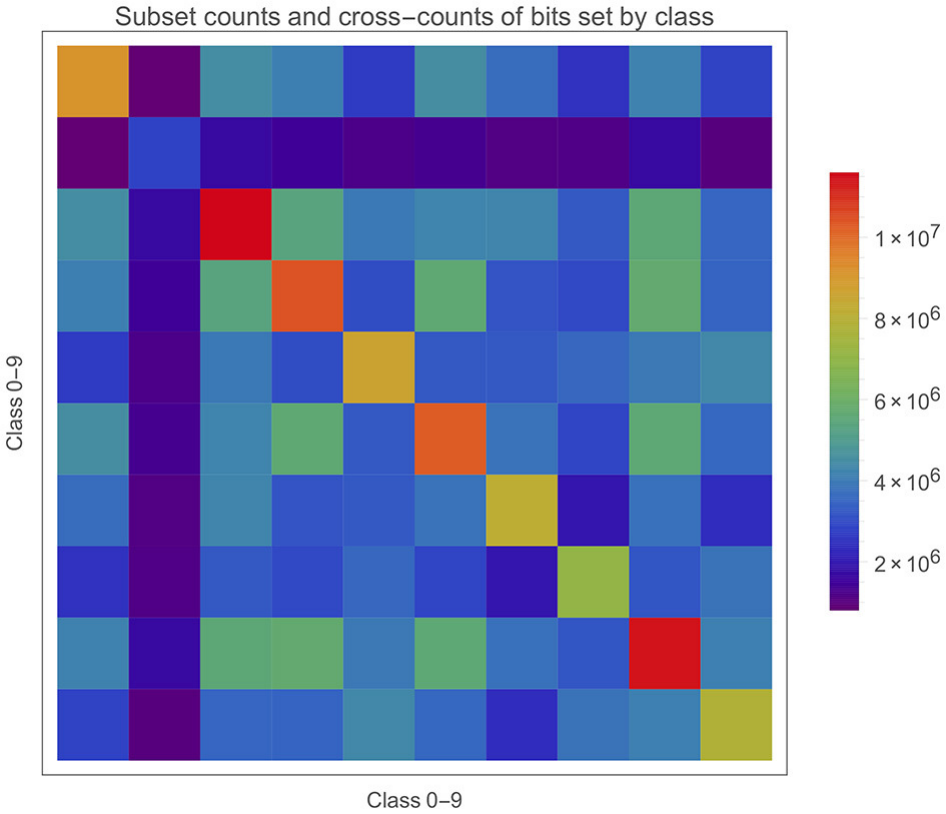
Cardinality of subsets and intersections for *length*(***b***) = 33.5M in this case.

It's also worth bearing in mind that the **S**{***b***_i_} will intersect with each other in potentially complex ways. An intersection between any number of **S**{***b***_i_} simply means the subset of memory positions in all the SBC memories where those class bits are all set. Figure 11 gives a matrix plot for the number of bits in each **S**{***b***_i_} on the diagonal and the two-way intersections between these subsets off the diagonal. The intersections are substantial, but does it matter or is it inevitable? What about three- and higher- (i.e., up to *c*-) way intersections?

We can observe the different cardinalities of each **S**{***b***_i_} on the diagonal which is particularly low for class **1** and high for classes **2** and **8**. However, directly correcting for this (e.g., a naive multiplication by their inverse ratio) makes prediction accuracy worse, and so the cardinality is not all that matters. One observation is that **1**s might be more similar, leading to fewer bits being set in **S**{***b***_1_} because the same bits are constantly being activated but cannot be stored more than once. The relatively low values of ***h***_i_ in Figure 10 for class **1** would tend to support this. This may not matter in inference if the test cases have a similar behaviours and are well-separated in feature space from the other classes. Presumably, “well-separated” is some function of the intersections (of various orders) between all the **S**{***b***_i_}.

For precise inference it would be ideal to have no intersections at all, but minimising them is a much more realistic aim. That would suggest a very clear difference in the bit count values between classes assuming that test cases follow the training case patterns, which is a realistic assumption that is often required in machine learning. Achieving this kind of behaviours where classes of interest are separated as widely as possible is a standard problem in optimal experimental or sampling design (Shewry and Wynn, 1987). However, working out how to get to that position in this problem is not easy. One would need to ensure that entirely different patterns of bits are set by the different classes which would mean consistently different combinations of ADEs firing per class, and it's not currently clear how this could be achieved. Some early work tried learning each $\frac{1}{10}$^th^ of the ADEs (i.e., the feature detectors) on digit data distribution separately by class or differences between classes at the unsupervised stage, but this did not provide any significant improvement in prediction performance.

To visualise the intersections of active bits in o_*_ with those of **S**{***b***_i_}, Table 3 shows the first results from the same setup as Figure 11. The number of active bits in the test case shows that the ADEs are tending to fire at slightly more than the 1% target rate, at least assuming complete randomness.

**Table 3:** The first results from the same setup as Figure 11 with classes {0, ... , 9}, *length*(***b***) = 33.5M.

***card*** (o*)	***class***	**0**	**1**	**2**	**3**	**4**	**5**	**6**	**7**	**8**	**9**
12,168	7	4,876	2,909	6,529	6,916	7,291	6,398	2,769	12,055	5,295	9,704
21,011	2	13,624	8,495	20,348	16,114	4,068	12,939	14,645	2,930	13,362	3,179
7,320	1	2,551	7,257	5,965	5,115	5,764	5,160	4,531	6,147	5,962	5,010
15,664	0	15,409	1,941	9,625	8,997	5,459	10,297	11,040	7,434	5,724	7,152
14,110	4	6,816	2,625	9,796	6,231	13,919	7,156	8,005	9,553	7,969	11,495
8,597	1	2,442	8,515	6,577	5,184	6,452	4,983	4,287	7,522	6,843	5,335
10,804	4	3,005	3,087	5,594	6,073	9,974	6,007	4,667	6,638	7,824	7,644

*card*(o^*^) is the number of active bits in the test case, *class* is the class for the test case, and the remaining columns show the intersections of the test case with each of the training classes.

There are several things of interest here. Firstly the correct class has been chosen each time (this sample is from a 97.12% performance, so not such a surprise). Secondly is that almost all the active bits in a test case intersect with the bits in the relevant **S**{***b***_i_}. Of course this is good but such a high overlap is somewhat surprising and this seem to be quite general across both test cases and classes. Thirdly, the size of overlap with the wrong classes is also much higher than expected. Let's take the first case for example and look at class **9** instead. The overlap with **S**{***b***_9_} = 9,704. *card* (**S**{***b***_9_} ) = 7,859,278 which gives as a proportion of all possible bits a 23.42% occupancy. There are 3,803,472 bits shared between **S**{***b***_7_} and **S**{***b***_9_} (see Figure 11) and *card*(**S**{***b***_7_}) = 7,075,762. So we can say that this two-way intersection is about half of each subset! In other words, for any memory position where one of these class bits has been set there is about a 50% chance that the other is set as well. **7** and **9** are likely to be a worse than average case as they are often one of the higher value pairs in the confusion matrix.

## 7. Discussion

In this section, we aim to cover some loose ends and discussion points that surround what is basically a simple idea that we have tried to describe in a straightforward way.

Although we have focused on the benefits of *BitBrain* some may wonder what is to be paid for these. One discussion has been about how much data needs to be stored in the SBC memories and that a comparison should be made with methods that use this amount of parameter space. We disagree with this perspective, for the following reasons. We see *BitBrain* as a non-parametric method. There is no definitive description of a non-parametric model but this is as good as any.^5^

Non-parametric machine learning algorithms try to make assumptions about the data given the patterns observed from similar instances. For example, a popular non-parametric machine learning algorithm is the K-Nearest Neighbor algorithm that looks at similar training patterns for new instances. The only assumption it makes about the data set is that the training patterns that are the most similar are most likely to have a similar result.

Another parallel is found here then with Kernel methods which—expressed in the form described in Section 6—are clearly non-parametric in nature as they use the data directly for their inference, not parameters that have been somehow inferred from the data. There may sometimes be hyperparameters involved (for example the local width of the kernel in Kernel regression) but nevertheless the predictor is formed from a relatively simple function of the data themselves.

*BitBrain* is clearly working in a similar way, and in fact the only scalar parameters learned are during the unsupervised learning stage where ADE thresholds are found which give an approximately correct average firing probability. These are local 1D searches that are cheap and easy to carry out in parallel and with a well-defined optimum point. There are also some differences, for example it is not the data *themselves* that are used but a high-dimensional projection of them *via* ϕ(). We don't believe this changes anything though in relation to the fundamentally non-parametric nature of the inference. So the SBC memories are storing “a direct encoding of the data in a form which makes it suitable for inference”, not “parameters”.

It should also be said that there are versions of the method with a much lower memory footprint. For example, if only memory positions containing a single class bit after supervised learning are saved. In the context of the discussion in Section 6 this means activated parts of the bit space where there are no intersections between class subsets. This excludes the vast majority of memory positions and also means that less space is needed for each. One may achieve 2-4 orders of magnitude saving in storage with a demonstrated small (≈3% on MNIST) loss of inference performance but also a significant gain in inference speed, though hash tables or some other mechanism will be needed for the required sparse storage unless specialised hardware is available and this will somewhat complicate the implementation. It may be that in some applications these trade-offs are fully justified.

Another unknown is the capability of the current and basic implementation on the most challenging problems. We are unlikely to compete with deep networks with billions or trillions of parameters at this point, but in defence of the method it was never designed for this. It remains to be seen how the more advanced versions discussed below fare in such problems.

### 7.1. Future directions

*BitBrain* is a very new method and there is a lot left to explore in order to understand and improve behaviours. In this subsection, we briefly describe a number of ideas that are either under consideration or being actively investigated.

It has been observed that various ADEs will tend to fire together more than they should by chance, whereas the ideal would appear to be independence amongst the ADE firing patterns. There are various arguments from both information theory and SDM theory to support this aim, though it is by no means proven. Some mechanism for modifying or replacing ADEs which are too similar would therefore appear to be a useful mechanism during the unsupervised learning phase. Earlier work in unsupervised learning may be relevant here (Atick and Redlich, 1993; Bell and Sejnowski, 1995; Linsker, 2005).

There can be several subtleties and variations for the basic supervised learning phase described in Section 4.7. We might, e.g.,

- Only write a bit probabilistically which will reduce memory occupancy and potentially increase inferential robustness.
- Add noise to the training data to facilitate inferential robustness (as seen in Figures 3–7).
- “Jitter” or otherwise augment the training data with elastic deformations and rotations.
- Use *N-of-M* codes within each memory location to specify the class encoding and decoding.
- Enforce a strict *N-of-M* code for each AD activation by choosing the *N* ADEs that exceed their threshold by the largest amount.

There will be weightings of the class bit sums which perform better than the default (uniform) case. The simplest weighting is to element-wise multiply by a vector the same length as the number of classes. A more flexible weighting which can take into account—and perhaps correct for—complex inter-correlation patterns between the classes is to post-multiply the class bit counts by a square matrix and use the resulting vector for class choice. In either of these cases, the challenge is to know what this vector or matrix should be!

Two types of continuous learning are being considered. Firstly, dealing with data that changes over time: perhaps the degradation of a sensor input device or a genuine change in patterns in the system of interest. As new labelled data arrives over time these can be added to the SBC memories as in the supervised phase. A count would be kept of the number of bits added and at a chosen interval this same number of bits are randomly removed from the SBCs. This will maintain the memory occupancy at the chosen level. Over time, even if the nature of the input data has diverged significantly from the original training set this very simple mechanism will adapt automatically to changing circumstances and retain predictive performance on the most recent inputs. The key decision is how quickly to adapt and this will differ for each problem. The second possibility is that of adding new classes, which apart from the administration of the SBC memories would be almost automatic. In both of these cases there may be an argument for another phase of unsupervised learning (or perhaps occasional updating) but this would not necessarily be required. Contrast the simplicity of these solutions with the problems facing most other ML methods.

An application where these ideas might be fruitfully applied is in the ML sensor 2.0 paradigm (Warden et al., 2022) which we feel is a natural fit for *BitBrain* for the following reasons:

- A very simple interface is required to provide security and engineering modularity.
- The impact on model building, training, software development and integration. For example, the speed with which a specific sensor could be taught its own custom model on a training set with *BitBrain* and thereby sidestep issues about production variability and the sharing of large and complex pre-trained models.
- Continuous learning within the black box will generally be a problem but not for *BitBrain*.
- Inference that is robust to sensor degradation and environmental variability is considered essential.
- Specific and neuromorphic H/W is seen as the future of such low energy/always on devices.

The unsupervised learning mechanism described above has been shown to work well, however there may be other approaches using work from image processing in ML which can provide a useful alternative mechanism. Convolutional neural nets have been shown to provide very good results on challenging problems and in many cases the convolutional front end can be reused across problems of a similar nature. This could allow us to go directly from acquiring the data to the single-pass learning mechanism, and at the same time sidestep the issue of needing to relearn the front end in continuous learning problems.

We have only discussed single channel image data in this paper. We are keen to expand beyond this into any type of data and preliminary results are very promising. For example:

- Multi-channel (such as RGB) image or volumetric data (e.g., in medical imaging).
- DNA, IP or other engineering/biological/pharmaceutical codes with no obvious locality structure.
- Uni- or multi-variate time series including real-time data from event-based sensors so that temporal as well as spatial patterns can be classified.

There are a number of very interesting questions to answer as we expand the technique into these areas.

Just as in other areas of AI and ML, layers of inference and/or hierarchies can be very powerful extensions of a basic learning and inference mechanism. We believe that the same may be true of *BitBrain*. The question will be: how to connect the SBCs together? For example, in forming layers of SBCs, we would need some form of ‘output' from the upper layer which is not the class itself. This would then feed the next layer as input. There are a number of interesting possibilities currently under consideration. This also provides the option that information flow can be feedback as well as feedforward, and therefore the opportunity for more end-to-end style learning mechanisms.

Finally, we are interested in experimenting with robustness in the presence of input perturbation of more realistic forms than simple Gaussian or Salt and Pepper noise added to the data.

There are so many possibilities here that separate papers will be required to address them all.

### 7.2. Neuromorphic interpretation

All computational operations for *BitBrain*—whether in training or inference—are small integer addition/multiplication or bitwise operations, which can be performed in one cycle on a RISC architecture with low energy use and often also leverage efficient SIMD units. This is rarely the case in typical ANN computations. The energy benefits of this difference will vary widely but an order of magnitude is not unrealistic.

While the *BitBrain* architecture can be mapped onto existing neuromorphic computational devices, there are likely to be greater gains in performance and energy-efficiency from mapping it into neuromorphic hardware specifically designed to support it. Figure 2 is highly suggestive of a possible hardware implementation of an SBC, comprising a 2D array of nodes where each node incorporates a number of SRAM cells to store the class bits. This 2D array has a row AD and a column AD, where each AD is a linear array of ADEs. The vector of input values is broadcast across all the ADEs, each of which selects its chosen inputs, computes its activation and, if this exceeds its threshold, fires its output. Wherever two active ADE outputs meet across the 2D array the corresponding node is activated for a read or write operation. Writing can use a class indicator broadcast across the 2D array; reading is trickier as it involves counting the 1s for each class across all of the activated nodes, which could be achieved using analogue techniques or perhaps by serially pulsing the active row ADEs and counting the 1s by column.

#### 7.2.1. *BitBrain* implementation on SpiNNaker

On some of the existing neuromorphic devices, such as Loihi and TrueNorth (Akopyan et al., 2015; Davies et al., 2018), the ADEs could potentially be mapped onto individual units resembling simple spiking neurons. The SpiNNaker system (Furber and Bogdan, 2020) offers a number of ways to directly take advantage of the sparsity and parallelism inherent in *BitBrain* thanks to its ability to execute non-neural simulations. SpiNNaker is a digital neuromorphic platform which can host up to 1 million general purpose processors in a single cluster, thus allowing for an extremely high number of calculations to be executed in parallel. The SpiNNaker platform, as well as the discussed implementation of the *BitBrain* algorithm, are designed to allow for robust and loss-tolerant routing of messages. Therefore, a loss of information from faulty nodes is redundant for the computation.

There are many different ways to implement such a system on SpiNNaker. In this section, we will discuss one such implementation which consists of two types of application vertices: ADE and SBC vertices. Here, an application vertex is understood as a particular type of application running on an independent core in the SpiNNaker system. These applications carry out certain computational tasks, and then communicate between each other *via* multicast packets which contain certain type of payload specific to the application. In the extremely parallel implementation which assumes a *BitBrain* instance with 4 ADs of length 2,048, the ADE vertices alone would require 8,192 cores to be employed. Additionally, 6 cores would be needed for the SBC vertices which receive firing patterns and interact with the SBC memory. We found that such massively parallel implementations are sub-optimal, due to the large number of messages which need to be processed. Moreover, we found that computation done by the SBC vertices requires more time to be completed, therefore further parallelisation of ADE processing does not bring expected gains in performance.

For the purpose of this paper we decided to focus on an implementation which parallelises not only the ADE calculations but also writing and reading from the SBC memories. Note that this in an early implementation and we plan to do a more thorough study to understand and explore the many options, as there is so much flexibility in how to implement the algorithm on SpiNNaker that finding the best balance between, e.g., data movement, AD calculations, SBC calculations, message types and quantity, memory access patterns is a large and sometimes counter-intuitive task. Moreover, the SpiNNaker architecture is based on rather old processors ARM9 with clock rate of 200 MHz.

**Figure 12:**
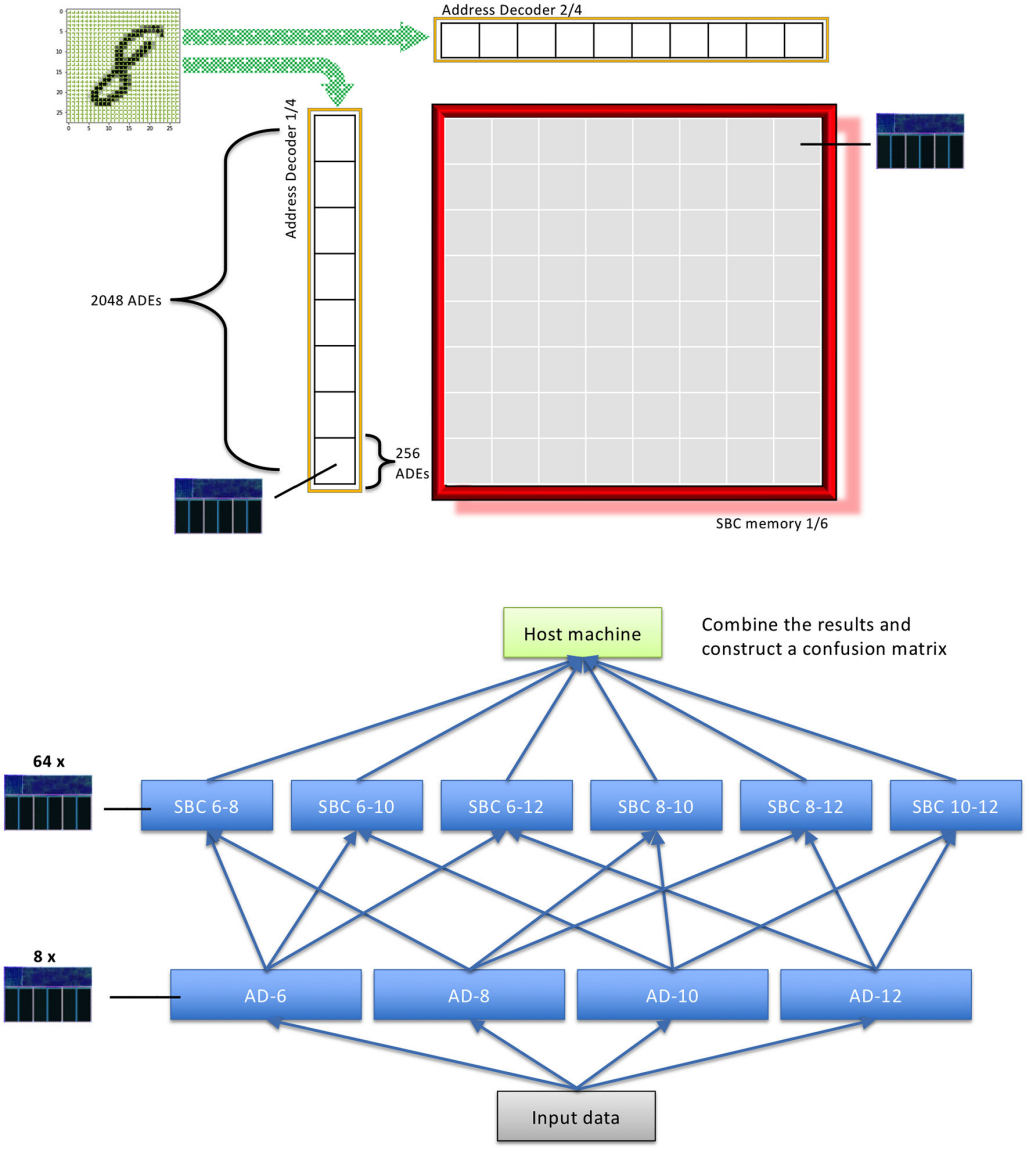
An example of distributed implementation of *BitBrain* on SpiNNaker neuromorphic platform. In this example, we divide the SBC computation into 64 parts per SBC memory. Additionally, each Address Decoder is split into 8 cores consisting of 256 ADEs. The panel below illustrates the message routing between different application vertex types in a parallelised implementation of *BitBrain*.

We found that it is the most beneficial to divide each SBC into 64 cores which only receive messages from a small subset of ADE vertices, see the upper panel of Figure 12. Thus, we use 384 cores in total for reading and writing from the SBC memory. We then divide the ADE computation into further 128 cores. With this approach we've been able to recreate the performance we have previously recorded on conventional processors, and achieve an inference time of approximately 48s, or ≈4.8 ms per data point. Notably, the performance accuracy of our implementation is higher than that of other approaches previously implemented on SpiNNaker, such as Liquid State Machines (LSM) (Patiño-Saucedo et al., 2022). However, it is important to emphasise that these previous attempt typically used a variant of MNIST dataset—Neuromorphic MNIST (Orchard et al., 2015), which requires additional preprocessing steps and operates on temporal inputs, thus cannot be compared directly.

The ADE cores are responsible for calculating the firing patterns of a small subset of pixels in the image. When activated, an ADE vertex sends a message to the set of its corresponding SBC vertices, see the lower panel of Figure 12. In turn, the SBC vertices collect the incoming messages, and calculate the feature coincidences. The updates to the application vertices are performed on a timer interrupt which occurs every simulation time step. The length of this time step is determined by the complexity of the calculations which need to be performed and he messages that need to be processed. On average the SBC vertices receive ≈60 activation messages from the ADE vertices, with an additional 16 messages which are meant to indicate that the ADE vertices have finished their part of the job. On the other hand, the ADE vertices receive 192 messages per one image in the dataset.

The training and testing sets, as well as other data structures required to implement a *BitBrain* instance are stored in two types of memory: SDRAM (shared slow memory) which contains the SBC memories and the full set of training/testing data, and DTCM (fast local memory) which contains the simulation parameters, routing keys, address decoder thresholds, firing patterns, and test labels (in inference mode). Additionally, we allow the ADE vertices to transfer the training/testing data for each example into the DTCM memory, while the system is waiting for the SBC vertices to interact with their respective SBC memories. This approach allows us to reduce the inference time further by approximately ≈25%.

Each of the SBC vertices has a recording channel. In the inference mode the count of SBC activations per class is being recorded for each example in the test set. In the training mode the SBC memories are recorded only once after the whole training set has been processed. The recordings are then accessed by the host machine in order to save the trained SBC memories, or calculate the inference accuracy and build a confusion matrix.

## 8. Conclusions

We have introduced an innovative working mechanism (the *SBC memory*) and surrounding infrastructure (*BitBrain*) based upon a novel synthesis of ideas from sparse coding, computational neuroscience and information theory that support single-pass learning, accurate and robust inference, and the potential for continuous adaptive learning. We have demonstrated the efficacy of these concepts on the MNIST and EMNIST benchmarks and shown that the proposed inference mechanism has very low training costs and is robust to noise.

Clearly these ideas are not yet fully developed, and theoretical advances as well as practical experience are likely to provide further gains in performance and efficiency. There are various ways that the mechanisms can be reconfigured, for example to reduce the SBC memory requirements by using an efficient compressed sparse matrix storage format, and/or by reducing the number of classes stored at each potential coincidence node.

Even at this early stage of development, *BitBrain* displays state-of-the-art performance in one-shot learning tasks combined with intrinsic robustness and fast inference. The Sparse Binary Coincidence memories upon which it is based may be large, but are simple bit arrays set to mark principal feature coincidences. This mechanism supports continuous on-line learning provided that the memories do not become over full, which may be ensured by incorporating some form of random “forgetting”.

## Data availability statement

The original contributions presented in the study are included in the article/supplementary material, further inquiries can be directed to the corresponding author.

## Author contributions

MH and SF created and carried out testing of the original ideas. EJ and JF produced the comparative CNN results. JF implemented *BitBrain* on the SpiNNaker hardware. All authors contributed to the article and approved the submitted version.

## Footnotes

^1^A patent GB 2113341.8 covering this technology was filed by MH and SF at the UKIPO on 17th September 2021.

^2^An implementation of this basic mechanism in self-contained C source code with the necessary data files for the example in Section 5.1 is available here: https://doi.org/10.48420/c.6331565.v1

^3^CNN A—https://github.com/jiuntian/pytorch-mnist-example and CNN B—https://github.com/ya332/Simple-CNN-for-MNIST

^4^For actual computation it is both faster and numerically more stable to decompose *K* (e.g., using the Cholesky decomposition) and then solve for *y*_i_ using a backsubstitution rather than forming the inverse explicitly and then multiplying. This decomposition only needs to be done once but it is O(*n*^3^) and so clearly for large training sets is a time-consuming linear algebra operation. The solutions to form each *h*_i_ are O(*n*^2^) and only need to be done once.

^5^ https://deepai.org/machine-learning-glossary-and-terms/non-parametric-model
